# Salivary MicroRNAs for Early Detection of Head and Neck Squamous Cell Carcinoma: A Case-Control Study in the High Altitude Mestizo Ecuadorian Population

**DOI:** 10.1155/2018/9792730

**Published:** 2018-11-21

**Authors:** Carolina Salazar-Ruales, Jessica-Viviana Arguello, Andrés López-Cortés, Alejandro Cabrera-Andrade, Jennyfer M. García-Cárdenas, Patricia Guevara-Ramírez, Patricio Peralta, Paola E. Leone, César Paz-y-Miño

**Affiliations:** ^1^Centro de Investigación Genética y Genómica, Facultad de Ciencias de la Salud Eugenio Espejo, Universidad UTE, Avenue Mariscal Sucre, 170129 Quito, Ecuador; ^2^Ingeniería en Biotecnología, Facultad de Ingeniería y Ciencias Agropecuarias, Universidad de las Américas, Avenue de los Granados, 170125 Quito, Ecuador; ^3^Hospital Oncológico Solón Espinosa Ayala, Avenue Eloy Alfaro, 170138 Quito, Ecuador

## Abstract

Head and neck squamous cell carcinoma (HNSCC) is the sixth most common cancer with the highest incidence worldwide. HNSCC is often diagnosed at advanced stages, incurring significant high mortality and morbidity. The use of saliva, as a noninvasive tool for the diagnosis of cancer, has recently increased. Salivary microRNAs (miRNAs) have emerged as a promising molecular tool for early diagnosis of HNSCC. The aim was to identify the differential expression of salivary miRNAs associated with HNSCC in the high altitude mestizo Ecuadorian population. Using PCR Arrays, miR-122-5p, miR-92a-3p, miR-124-3p, miR-205-5p, and miR-146a-5p were found as the most representative ones. Subsequently, miRNAs expression was confirmed in saliva samples from 108 cases and 108 controls. miR-122-5p, miR-92a-3p, miR-124-3p, and miR-146a-5p showed significant statistical difference between cases and controls with areas under the curve (AUC) of 0.73 (p < 0.001), 0.70 (p < 0.001), 0.71 (p = 0.002), and 0.66 (p = 0.008), respectively. miRNAs were also deregulated in between HNSCC localizations. A differentiated expression of miR-122-5p between oral cancer and oropharynx cancer (AUC of 0.96 p = 0.01) was found: miR-124-3p between larynx and pharynx (AUC = 0.97, p < 0.01) and miR-146a-5p between larynx, oropharynx, and oral cavity (AUC = 0.96, p = 0.01). Moreover, miR-122-5p, miR-124-3p, miR-205-5p, and miR-146a-5p could differentiate between HPV+ and HPV- (p=0.004). Finally, the expression profiles of the five miRNAs were evaluated to discriminate HNSCC patient's tumor stages (TNM 2-4). miR-122-5p differentiates TNM 2 and 3 (p = 0.002, AUC = 0.92), miR-124-3p TNM 2, 3, and 4 (p < 0.001, AUC = 98), miR-146a-5p TNM 2 and 3 (p < 0.001, AUC = 0.97), and miR-92a-3p TNM 3 (p < 0.001, AUC = 0.99). Taken together, these findings show that altered expression of miRNAs could be used as biomarkers for HNSCC diagnosis in the high altitude mestizo Ecuadorian population.

## 1. Introduction

Head and neck squamous cell carcinoma (HNSCC) is the sixth most common cancer with the highest incidence in the world, with 600,000 new cases per year and a mortality rate between 223,000 and 300,000 deaths [1–5]. The overall incidence of HNSCC is 8.8 and 5.1 cases per 100,000 inhabitants in men and women, respectively [1, 5]. The tumors originate in the epithelial cells of the mucosal linings of the upper airway and food passages (oral cavity, oropharynx, larynx, and pharynx) [6]. Oral cancer is the most common HNSCC. The primary screening test for oral cancer is a systematic examination, including inspection and palpitation of the oral cavity [7]. The highest incidence of oral cavity cancer is found in Southern and Central Asia, Melanesia, Southwestern Europe, and Southern Africa [4]. On the other hand, serum Epstein Barr Virus- (EBV-) associated antibodies and circulating cell-free EBV DNA testing have been used for nasopharyngeal cancer diagnosis and screening [8]. This type of cancer has a higher incidence in Southern China [4]. Additionally, there is no screening test to find early laryngeal cancer at the moment. The only way to screen this type of cancer would be a test called flexible endoscopy, taking a biopsy from the lining of the larynx. Laryngeal cancer has a higher incidence in Southern and Eastern Europe, South America, and Western Asia [4, 9]. In Ecuador, HNSCC is the third most frequent cancer for women and sixth for men, with 1637 new cases per year, being larynx cancer the most common one [10].

The risk factors for developing HNSCC include tobacco consumption, either smoking or chewing, and alcohol consumption. Both of these factors account for nearly 90% of the cases and are associated with age, sex, and ethnicity [11, 12]. Additionally, the Human Papillomavirus (HPV) infection is a risk factor for oropharyngeal cancer and EBV infection for nasopharyngeal cancer. The relative frequency of these risk factors contribute to the variation in the observed distribution of HNSCC worldwide [3, 9].

HNSCC is often diagnosed at advanced stages (T3 or T4) when survival rate is reduced to 20%, incurring significantly high mortality and morbidity [3, 13, 14]. On the other hand, the 5-year survival rate is 80%, if detected at early stages (T1 or T2). Therefore, the availability of biomarkers of different racial/ethnic populations with a potential application for HNSCC screening, early diagnosis, and monitoring of response to therapy are indispensable in clinical practice nowadays [12, 15].

The use of ‘liquid biopsies,' such as saliva, for the analysis of biomarkers might predict relapse at the earliest stage [6]. Saliva analysis has recently increased as a noninvasive and inexpensive tool for cancer diagnosis. Saliva has advantages at the time of collection, transport, and processing, compared to blood or tissue samples [16, 17]. In addition, salivary transcriptome has been reported with more than 3,000 species of RNAs and microRNAs (miRNAs) that can be used as biomarkers for the noninvasive diagnosis of HNSCC [3, 18–20]. According to Koshizuka et al., 2016, there are several studies of downregulated and upregulated miRNA expression profiles in HNSCC specially located in hypopharyngeal, larynx, oropharynx, maxillary sinus, oral cavity, and nasopharyngeal [21–31]. Hence, miRNAs have emerged as a promising molecular tool for diagnosis, prognosis, and therapy of HNSCC. miRNAs are currently defined as a small noncoding RNA type of 19 to 25 nucleotides in length, which regulate gene expression at the posttranscriptional level by blocking translation or inducing degradation of messenger RNA [32]. Multiple genes are regulated by a single miRNA, and multiple miRNAs can regulate a gene [6]. The specificity and biological properties of miRNAs make them a potential biomarker in the pathogenesis of cancer. miRNAs are expressed intracellular and extracellular in many tissues such as saliva, an easily accessible and noninvasive fluid with high stability [3]. Specific miRNAs and their expression pattern have been established in different primary tumors in HNSCC [33]. Zahran et al., 2015, identified three salivary miRNAs as potential markers of malignant transformation in oral mucosal lesions [34]. The aim of this study was to identify the differential expression of salivary miRNAs in order to develop a noninvasive and inexpensive tool for the diagnosis of HNSCC in the high altitude mestizo Ecuadorian population.

## 2. Materials and Methods

### 2.1. Study Subjects

All 216 individuals belong to the high altitude mestizo Ecuadorian population (2,800 meters above sea level). Unstimulated whole mouth resting saliva was collected from 108 HNSCC patients (stages 1 to 4), from the* Hospital Oncológico Solón Espinosa Ayala*, Quito, Ecuador, between the years 2015 and 2016. Demographic and clinicopathological characteristics of patients are listed in Table 1. Likewise, as for the control group, saliva samples were collected from 108 healthy individuals who were nonsmokers, with good oral hygiene and with no history of the disease. All samples were immediately processed in the laboratory. The Bioethics Committee at the* Universidad de las Americas* previously approved this study (No. 2013-1101). All participants provided written informed consent before sample collection.

### 2.2. Total RNA and miRNA Extraction

Total RNA from 200 *μ*L of saliva of 10 healthy controls and 10 patients was extracted using TRIzol, following manufacturer's instructions (Invitrogen, CA, USA). Total RNA was used to perform profiler PCR Arrays (miScript miRNA PCR Array) (Qiagen, Valencia, CA, USA), selecting the most representative miRNA in the Ecuadorian HNSCC population. In order to validate the selected miRNAs from the PCR Arrays, miRNAs were extracted from 300 *μ*L of saliva from all 216 participants using mirVana™ miRNA Isolation kit (ThermoFisher Scientific, CA, USA), according to the manufacturer's instructions. miRNAs were eluted in 30 *μ*L of RNase free water. Total RNA and miRNA concentrations were measured using a NanoDrop 2000 (ThermoScientific, Waltham, MA, USA) and samples with OD 260:280 ratios ≥1.8 were included in the study.

### 2.3. Total RNA and miRNA cDNA Synthesis

The complementary DNA (cDNA) synthesis from total RNA was carried out using the RT^2^ First Standard Kit (Cat. No. 330401) (Qiagen Sciences, Maryland 20874, USA). Prior to cDNA synthesis, a genomic elimination reaction was performed, according to manufactures instructions. Briefly, 2 *μ*L of GE buffer were added to 400 ng of RNA in a final reaction volume of 10 *μ*L. The reactions were incubated 5 min at 42°C in a SureCycler 8800 (Agilent, Santa Clara, CA, USA) and then kept on ice for 5 min. For the reverse transcription, all the genomic DNA elimination mix was converted into cDNA adding 10 *μ*L of the reverse transcription mix and incubated for 15 minutes at 42°C and 5 minutes at 95°C. Then 91 *μ*L of RNase free water were added to the reactions and mixed by pipetting. The reactions were kept at -20°C until the development of the real-time PCR Arrays. miRNA cDNA synthesis was performed with miScript II RT cDNA synthesis kit using Hispec buffer (Qiagen, Valencia, CA, USA), following the manufacturer instructions.

### 2.4. miRNA Profiler PCR Arrays

To select the most representative miRNAs in the high altitude mestizo Ecuadorian population affected with HNSCC, miScript miRNA PCR Array (Cat. No. MIHS-102Z) (Qiagen, Valencia, CA, USA) was used, according to manufacturer's instructions. First, cDNA from the total RNA, containing miRNAs, of 10 patients and 10 controls were diluted 1:10 in RNase free water. The miRNAs expressions were run in the Mx3005p qPCR System (Agilent, CA, United States) with the following cycling conditions: 95°C for 15 min, followed by 40 cycles of 94°C for 15 s, 55°C for 30 s, and 70°C for 30 s.

### 2.5. Relative miRNA Expression and the Comparative Livak Method (2^-ΔΔCT^)

Once the miRNA PCR Array was done, we calculated the relative miRNA expression using SNORD96A as the housekeeping normalizer through the Livak method (2^−ΔΔCT^) [35]. Relative quantification relates the PCR signal of the target transcript in a treatment group to that of another sample such as an untreated control [35]. Ct values >35 were excluded and miRNAs were selected based on fold-change >2 and p < 0.05, and p values were calculated based on a Student's t-test of the replicate 2^−ΔΔCT^ values for each miRNA in the case and control groups. Primers used for the selected miRNAs are the following: hsa-miR-92a-3p (UAUUGCACUUGUCCCGGCCUGU), hsa-miR-205-5p (UCCUUCAUUCCACCGGAGUCUG), hsa-miR-124-3p (UAAGGCACGCGGUGAAUGCC), hsa-miR-122-5p (UGGAGUGUGACAAUGGUGUUUG), hsa-miR-146a-5p (UGAGAACUGAAUUCCAUGGGUU), and SNORD96A (CCUGGUGAUGACAGAUGGCAUUGUCAGCCAAUCCCCAAGUGGGAGUGAGGACAUGUCCUGCAAUUCUGAAGG).

### 2.6. miRNA Quantitative Real-Time PCR

miRNA expression was confirmed by RT-qPCR. Saliva of 108 patients and 108 controls was analyzed with miScript SYBR Green PCR kit (Qiagen, Valencia, CA, USA). RT-qPCR was run in triplicates in a Mx3005p qPCR System (Agilent, CA, United States) with the following conditions: 95°C for 15 min, 40 cycles of 94°C for 15 s, 55°C for 30s,and 70°C for 30s. Ct values >35 were excluded, and 2^−ΔΔCT^ method was used to determine fold-changes.

### 2.7. Statistical Analysis

Relative quantification method was used to compare differences between patients and controls. The data were analyzed with miScript miRNA PCR Array Data Analysis Excel web portal (https://dataanalysis.sabiosciences.com/pcr/arrayanalysis). Additionally, Mann–Whitney U test for nonparametric analysis was applied to compare miRNA expression levels after Livak method [35]. Finally, in order to determinate the utility of the miRNAs as diagnostic biomarkers, receiver-operating characteristics (ROC) curves were generated. Ct values were normalized using SNORD96A as housekeeping gene. All statistical analyses were performed using the Graph Pad Prism 7 software version 7.02 (Graph Pad Software Inc., USA).

## 3. Results

Out of the 108 patients, 56 were females and 52 males; the median age was 53.5 (26-81) years old. 28.57% of patients were regular smokers (≥ 1 cigarette per day); out of those 3.9% were females and 26.7% males. Alcohol consumption was higher in males (14.3%) than females (6.7%). Moreover, 12.7% of patients were HPV positive (HPV+), 54.5% had tumor stage (TNM) 2, 30.9% TNM 3 and 14.5% TNM 4 (Table 1).

**Figure 1:**
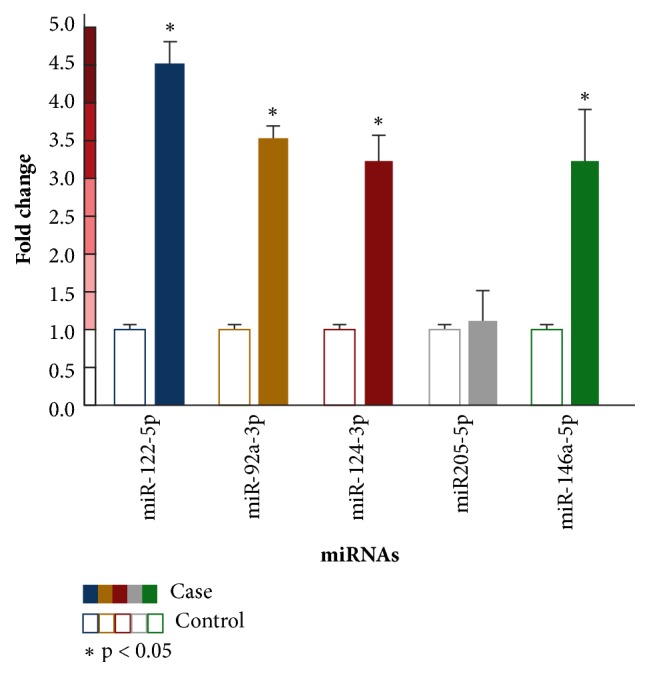
Average of relative expression of salivary miRNAs levels determined by RT-qPCR. miRNAs were normalized with the SNORD9a housekeeping gene. Case bars show the average of fold-changes of 108 affected individuals per miRNA. Control bars show the relative fold-change (n = 1) of 108 healthy individuals, according to the Livak method. Only miR205-5p was considered nonsignificant (p > 0.05).

From the PCR Arrays, miR-122-5p, miR-92a-3p, miR-124-3p, miR-205-5p, and miR-146a-5p were selected as the most representative ones in the Ecuadorian HNSCC population based on fold-change >2 (p < 0.05). miR-122-5p and miR-146a-5p were upregulated while miR-92a-3p, miR-124-3p, and miR-205-5p were downregulated (fold-changes 11.5, 10.79, 11.24, 25.51, and 55.7, respectively). Subsequently, expression of the five selected miRNAs was confirmed in saliva samples from 108 patients and 108 controls. Interestingly, all the miRNAs studied showed regulation in the fold-change values, miR-122-5p: 4.47 (p < 0.05), miR-92a-3p: 3.50 (p < 0.05), miR-124-3p: 3.18 (p < 0.01), and miR-146a-5p: 1.12 (p < 0.01) (Figure 1), showing a statistical difference between the miRNAs in patients and controls with the exception of miR-205-5p (p > 0.05).

In order to determinate the discriminatory differentiation of miR-122-5p, miR-92a-3p, miR-124-3p, and miR-146a-5p, receiver operator characteristic (ROC) curves were generated. The four miRNAs showed significant statistical difference with AUC of 0.73 (p < 0.001), 0.70 (p < 0.001), 0.71 (p < 0.05), and 0.66 (p < 0.01), respectively.

**Figure 2:**
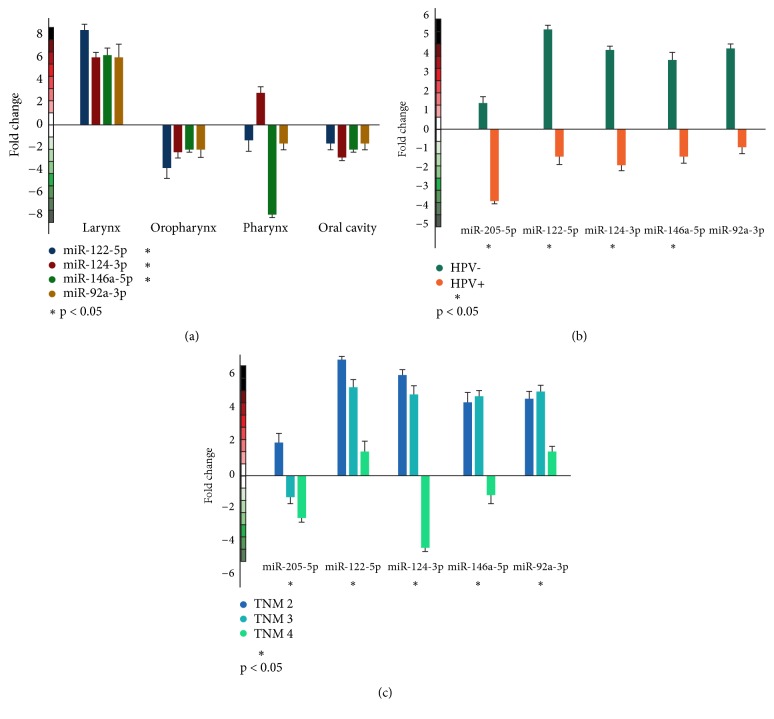
Relative expression levels of miRNAs in affected individuals. (a) Relative miRNAs expression, according to the HNSCC tumor location. (b) Relative miRNAs expression, according to the HPV status. miR-122-5p, miR-124-3p, miR-205-5p, and miR-146a-5p could differentiate between HPV+ and HPV- patients. (c) Relative miRNAs expression, according to TNM stage.

The ability of miRNAs to differentiate among HNSCC localizations was also evaluated (Figure 2(a)). miR-122-5p was upregulated in patients with HNSCC in larynx (fold-change of 8.27, p < 0.05) and downregulated in oropharynx HNSCC patients (fold-change of 3.83, p < 0.01), AUC of 0.96 p < 0.01. Moreover, an upregulation was found in miR-124-3p and patients with larynx and pharynx cancer (fold-changes of 5.91 and 2.77, respectively, p < 0.001). On the other hand, a downregulation was found in oropharynx and oral cavity patients (fold-changes of 2.03 and 3.9, respectively, p < 0.01). A differentiation among these localization sites was done with AUC of 0.97. miR-146a-5p could differentiate patients with HNSCC in the larynx, oropharynx, pharynx, and oral cavity (AUC of 0.96 p < 0.01) with upregulation in the larynx (fold-change of 5.98) and downregulation in the oropharynx, pharynx, and oral cavity (fold-changes of 2.21, 7.88, 2.2, respectively, p < 0.01). miR-92a-3p showed no significant difference (p > 0.05) to distinguish HNSCC localizations.

Additionally, we also evaluated the differentiation power of the miRNAs between HPV+ and HPV- patients (Figure 2(b)). Interestingly, in HPV+ patients all miRNAs were downregulated and in HPV- patients all miRNAs were upregulated. The relative expression between HPV+ and HPV- was significant in miR-205-5p, miR-122-5p, miR-124-3p, and miR-146a-5p (p < 0.05). However, miR-92a-3p showed no significant difference (p > 0.05).

Finally, the expression profiles of the five miRNAs were evaluated to discriminate HNSCC patient's tumor stages (TNM 2-4) (Figure 2(c)). miR-122-5p could differentiate TNM 2 and 3 (p < 0.01) with AUC of 0.92. miR-124-3p was able to differentiate stages 2, 3, and 4 (p < 0.001) and AUC of 0.98. miR-146a-5p differentiates between TNM 2 and 3 (p < 0.001), AUC of 0.97. Finally, miR-92a-3p could differentiate HNSCC patients on TNM 3 (p < 0.001, AUC of 0.99).

## 4. Discussion

The increased incidence of HNSCC patients contrasts with the lack of early diagnosis biomarkers that reduce cancer-related morbidity and mortality rates [36]. miRNA profiles have been shown as potentially useful biomarkers in cancer diagnosis [36–40]. Different miRNA expression has been identified in several studies of different racial/ethnic populations [3, 19, 34, 36, 37, 41], and the association of miRNA profiles with the population genetic background could increase the understanding of the molecular mechanisms of HNSCC [42]. Several studies worldwide have identified downregulated and upregulated miRNAs as biomarkers for different tumor locations of HNSCC. Kikkawa et al., 2010, identified miRNAs in hypopharyngeal [21]. Hui et al., 2010, identified miRNAs in larynx, oropharynx, and hypopharynx [22]. Liu et al., 2010, identified miRNAs in head and neck [24]. Nohata et al., 2011, identified miRNAs in maxillary sinus [25]. Lajer et al., 2011, identified miRNAs in oral cavity [26]. Severino et al., 2013, identified miRNAs in oral cavity [27]. Fukumoto et al., 2014 and 2015, identified miRNAs in hypopharyngeal [28, 43]. Zhang et al., 2014, identified miRNAs in larynx [29]. Martinez et al., 2015, identified miRNAs in head and neck [30]. Wang et al., 2016, identified miRNAs in nasopharyngeal [31]. Finally, Lubov et al., 2017, have demonstrated that several studies in Caucasian and Asian populations showed elevated expression of miRNAs associated with poor prognosis in HNSCC and decreased expression of miRNAs correlated with lower survival and metastasis [13].

Analysis of ‘liquid biopsies' is being evaluated as a tool in the care of patients with cancer in order to predict relapse at the earliest stage [6, 44]. For instance, plasma EBV DNA is a biomarker for nasopharyngeal carcinoma [8]. Over the last decade, saliva has emerged as an alternative diagnostic medium to detect both local and systemic events. Saliva medium carries a number of advantages compared with other tissue testing. First, it is produced and collected locally near HNSCC malignancies. Second, saliva sample is noninvasive and easy to perform. Third, the readily available nature of saliva collection also permits multiple samples to be collected for validation and follow-up [45]. The detection of salivary miRNAs is a promising noninvasive method to determine the risk of premalignant head and neck lesions [46]. Park et al., 2009, identified miR-125a and miR-200a as downregulated miRNAs in oral squamous cell carcinoma [19]. Liu et al., 2012, identified miR-31 as upregulated miRNA in oral squamous cell carcinoma [32, 47]. Salazar et al., 2014, developed a panel of saliva-based diagnostic biomarkers for the detection of HNSCC independently validated, using miRNA expression data from The Cancer Genome Atlas (TCGA). They concluded that the saliva-derived miRNAs miR-9, miR-134, and miR-191 may serve as novel biomarkers to reliably detect HNSCC [3].

Regarding our study, we have studied the expression of five different miRNAs (miR-122-5p; miR-92a-3p; miR-124-3p; miR-205-5p; and miR-146a-5p), selected from a miRNA Profiler PCR Arrays, in 108 saliva samples of HNSCC patients and 108 controls, and examined the association with clinicopathological features to determine whether these miRNAs are useful as diagnostic biomarkers.

Previous studies describe miR-92a as a microRNA belonging to the miR-17 ~92 group, which was described as oncogenic. However, subsequent studies have analyzed the miRNAs belonging to this group, indicating that not all act as oncogenes [48]. A possible link between the differentiated expression of miR-92a and tumorigenesis in plasma of patients with different types of malignant neoplasia has been demonstrated. In leukemia this miRNA has been identified as an effective biomarker to distinguish between healthy patients and individuals [49]. Different expression patterns have also been identified in patients with colorectal cancer [50]. In addition, in patients with hepatocellular cancer it was observed that miR-92a had a downregulation compared to healthy controls [51]. A similar result was obtained in the Brazilian population where it was verified that the miR17 ~92 group had different expression (downregulation) in patients with HNSCC, different from our study where this miRNA was found upregulated. However, MacLellan et al., 2012, found an upregulation of miR-92a-3p in serum of HNSCC patients [52]. Moreover, several experiments have found miR-92a acting on the proliferation of cancer cells and tumor growth, inhibiting cell apoptosis and metastasis [53].

On the other hand, it has been shown that miR-124 appears as a tumor suppressor, and the low expression of this miRNA can trigger tumor initiation or progression [54]. Moreover, miR-124 was found reduced in patients with HNSCC, and it was seen that miR-124 binds to the 3′-UTR region of transcription to negatively regulate ESX levels (Epithelial Restriction with Serine) responsible for regulating the epidermal growth factor receptor in HNSCC [55]. However, according to Liu et al., 2010, miR-124 is downregulated in HNSCC patients [24], contrary to Wong et al., 2008, who found that miR-124 was upregulated in HNSCC patients [56], similar to our results. Additionally, Sethi et al., 2014, found that miR-124-3p works as a potential tumor-suppressor miRNA [32].

Regarding miR-122-5p, a study by Ma et al., 2010, showed that overexpression of miR-122 could induce apoptosis and arrest of cancerous cell development through reduced expression of the* Bcl-W* and/or* CCNG1* genes. These findings suggest that miR-122 may behave as a tumor suppressor in different types of cancer [57]. In a previous study, it was also shown that miR-122 plays an important role in the inhibition of tumorigenesis through regulation of the PI3K/Akt/mTOR/p70S6K pathway [58]. However, studies on this particular miRNA are limited, so this finding could define miR-122 as a specific biomarker for the diagnosis of HNSCC, empowering pharmacogenomics and precision medicine in the high altitude Ecuadorian mestizo population [12, 59–64].

miR-146a has been extensively characterized in head and neck cancer from several studies. In thyroid cancer miR-146a has been found to have a 3.9 fold-change lower in patients suffering from the disease compared to healthy individuals [65]. Another study showed that there is a relationship between miR-146a overexpression and progression to metastatic tumors in patients with nasopharyngeal cancer [56]. On the other hand, the involvement of miR-146a in carcinogenesis is demonstrated by its altered expression in several types of tumor, such as thyroid, prostate, pancreatic, gastric, and oral cancer [66]. miR-146a acts by suppressing the expression of the* kinase-1 *associated with the* interleukin-1* receptor, implicated in the* NF-κB* pathway, which has been shown to participate in human carcinogenesis [67]. Orsos et al., 2013, found that miR-146-5p increases the risk of developing HNSCC when it is upregulated [66], similar to our results.

It has been described that miR-205 is a tissue-specific miRNA highly expressed in the epidermis being essential both in epithelial biogenesis and in the maintenance of the epithelium; a low regulation can lead to alterations, such as the proliferation of cancer cells [68, 69]. In several studies miR205 has been shown to be a promising biomarker in HNSCC, with high sensitivity and specificity [70]. This is also confirmed in a study by Childs et al., 2009, who concluded that miR-205 appears to act as a tumor suppressor in HNSCC, and the reduced expression of this miRNA should result in increased expression of target oncogenes [71]. However, we did not find a statistical difference with this miRNA between patients and controls.

In this study, we have identified a panel of four biomarkers for the diagnosis of HNSCC (hsa-miR-92a-3p, has-miR-124-3p, hsa-miR-122-5p, and hsa-miR-146a-5p) in the high altitude mestizo Ecuadorian population. However, different populations with HNSCC show a differential expression of miRNAs. For example, Li et al., 2009, identify, in Chinese patients with HNSCC, that an increased expression of miR-21 leads to a decrease in the expression of phosphatidylinositol-3,4,5-trisphosphate 3-phosphatase (PTEN). In addition, when miR-31 is deregulated in different types of cancer, including head and neck cancer, the hypoxia-inducible factor (HIF) is activated, promoting tumor angiogenesis [72]. According to Xie et al., 2013, miR-144, miR-21, miR-451, miR-634, miR-486-5p, miR-10b, and several regulatory miRNAs were found in HNSCC patients; miR-10b, miR-144, and miR-451 were found in whole saliva and miR-10b, miR-144, miR-21, and miR-451 in the saliva supernatant. With this study it was possible to establish the presence of miRNAs in all salivary fractions for the detection of esophageal cancer [73].

Moreover, the deregulation of miRNAs in different TNM in HNSCC has been found. According to Chang et al., 2013, both miR-17 and miR-20a are downregulated in advanced TNM in oral squamous cell carcinoma [74]. We found that miR-205 was downregulated in 4 TNM patients, mir-122 upregulated in TNM 2 and 3, miR-124 upregulated in TNM 2 and 3 and downregulated in TNM4, miR-146 upregulated in TNM 2 and 3, and finally miR-92a upregulated in TNM 2 and 3. Furthermore, miR-146, -149, -196, and -499 were found to increase the risk of HNSCC associated to HPV16 [32, 75]. This is similar to our findings, where miR-146 was upregulated in HPV+ patients.

In conclusion, the increased incidence of HNSCC patients contrasts with the lack of early diagnosis biomarkers that could reduce cancer-related morbidity and mortality rates worldwide. miRNA profiles have been shown as potentially useful biomarkers in cancer diagnosis. Our findings have shown that altered expression of miRNAs is different from that one shown in other populations worldwide. That is, hsa-miR-92a-3p, has-miR-124-3p, hsa-miR-122-5p, and hsa-miR-146a-5p miRNAs could be promising biomarkers for HNSCC diagnosis in the high altitude mestizo Ecuadorian population who lives 2,800 meters above sea level.

## Figures and Tables

**Table 1:** Demographic and clinicopathological characteristics of patients with HNSCC.

**Gender**	**n**	**Smokers** **(**%**)**	**Alcohol consumption** **(**%**)**	**HPV status (**%**)**	**Tumor stage (**%**)**		
				**Positive**	**2**	**3**	**4**
Female	56	3.9	6.7	9.1	31.2	22.7	12.7
Male	52	24.7	14.3	3.6	23.4	8.3	1.8
All cases	108	28.6	37.5	12.7	54.6	30.9	14.5

HPV, Human Papillomavirus.

## Data Availability

The data used to support the findings of this study are included within the article.
